# An Optimized Collagen-Fibrin Blend Engineered Neural Tissue Promotes Peripheral Nerve Repair

**DOI:** 10.1089/ten.tea.2017.0457

**Published:** 2018-09-01

**Authors:** Christina M.A.P. Schuh, Adam G.E. Day, Heinz Redl, James Phillips

**Affiliations:** ^1^ Department for Nerve Regeneration, Ludwig Boltzmann Institute for Experimental and Clinical Traumatology, Vienna, Austria.; ^2^ Austrian Cluster for Tissue Engineering, Vienna, Austria.; ^3^ Consorcio Regenero/Cells for Cells, Universidad de Los Andes, Faculty of Medicine, Laboratory of Nano-Regenerative Medicine, Santiago, Chile.; ^4^ Department of Biomaterials & Tissue Engineering, University College London, UCL Eastman Dental Institute, London, United Kingdom.; ^5^ Department of Pharmacology, University College London, UCL School of Pharmacy, London, United Kingdom.

**Keywords:** engineered neural tissue, fibrin, collagen, nerve regeneration

## Abstract

Tissue engineering approaches in nerve regeneration often aim to improve results by bridging nerve defects with conduits that mimic key features of the nerve autograft. One such approach uses Schwann cell self-alignment and stabilization within collagen gels to generate engineered neural tissue (EngNT). In this study, we investigated whether a novel blend of fibrin and collagen could be used to form EngNT, as before EngNT design a beneficial effect of fibrin on Schwann cell proliferation was observed. A range of blend formulations was tested in terms of mechanical behavior (gel formation, stabilization, swelling, tensile strength, and stiffness), and lead formulations were assessed *in vitro*. A 90% collagen 10% fibrin blend was found to promote SCL4.1/F7 Schwann cell viability and supported the formation of aligned EngNT, which enhanced neurite outgrowth *in vitro* (NG108 cells) compared to formulations with higher and lower fibrin content. Initial *in vivo* tests in an 8 mm rat sciatic nerve model using rolled collagen-fibrin EngNT rods revealed a significantly enhanced axonal count in the midsection of the repair, as well as in the distal part of the nerve after 4 weeks. This optimized collagen-fibrin blend therefore provides a novel way to improve the capacity of EngNT to promote regeneration following peripheral nerve injury.

## Introduction

Peripheral nerve injuries affect ∼300,000 people annually in Europe and are a major burden to patients and social and healthcare systems due to frequent hospitalization, pain, and resulting disabilities.^1^ Although peripheral neurons have notable regenerative potential, regeneration over nerve gaps or over long distances faces several challenges. Transplantation of autologous nerve tissue is considered the current clinical gold standard; however, autografts are limited in availability and prone to unsatisfactory success rates, as well as side effects such as donor site morbidity.^1–5^

As an alternative, the use of artificial conduits, as well as decellularized allografts, to restore continuity between the proximal and distal stumps of a transected nerve has been the subject of a large number of investigations. Some of these approaches are currently used in clinical nerve repair, although there is an ongoing debate concerning their appropriate use, effectiveness, and side effects.^6,7^ One of the reasons for the unsatisfactory outcome after repair of long-distance gaps is the limited proliferative capacity of Schwann cells.^8,9^ Schwann cells play a key role in peripheral nerve regeneration as after injury they switch into a regenerative phenotype and proliferate to form the bands of Büngner, generating a highly aligned environment for the support and guidance of axons.^10,11^ Therefore, a biomimetic tissue-engineered equivalent growth substrate to deliver regenerative Schwann cells should ideally (1) maintain the proliferative status of Schwann cells for Büngner band formation and (2) induce and maintain alignment of Schwann cells for directional axonal outgrowth. The biomaterials collagen and fibrin have both been used for decades in attempts to improve peripheral nerve injuries as luminal fillers and substrates for Schwann cells.^12,13^ Previously we developed engineered neural tissue (EngNT), in which self-alignment of Schwann cells within a collagen type I hydrogel, followed by stabilization of the aligned structure by removal of some interstitial fluid, results in a tissue-like material suitable for nerve tissue engineering.^14^

Fibrin, its formation and degradation, is one of the major components in wound healing; not only in hemostasis but also as a provisional growth matrix for a variety of tissue specific cells.^15^ Especially in nerve regeneration fibrin plays a crucial role: after injury the nerve stumps leak fibrinogen plasma exudate into the affected area, resulting in fibrin cables that can support the migration of Schwann cells from the stumps.^1,16^ Furthermore, fibrin strongly induces the regenerative phenotype of Schwann cells by upregulating p75NGFR through phosphorylation of ERK1/2,^17^ but its rapid degradation impedes the use as a luminal filler for long gaps.^18,19^

The aim of this study was to investigate whether a collagen-fibrin blend could be used to form EngNT and to test the hypothesis that combining the Schwann cell-activating characteristics of fibrin with the stable structural properties of collagen in EngNT would result in an improved material for delivering aligned Schwann cells to a site of nerve injury. Mechanical properties, Schwann cell viability, phenotype, and support of neurite outgrowth were evaluated *in vitro*, allowing an optimal formulation of collagen-fibrin blend EngNT to be identified and then tested *in vivo* using a rat sciatic nerve repair model.

## Materials and Methods

Unless indicated otherwise, all reagents were purchased from Sigma-Aldrich and were of analytical grade.

### Evaluation of Schwann cell behavior on fibrin and collagen substrates *in vitro*

#### Primary rat Schwann cell isolation and culture

All animals were euthanized according to established protocols, which were approved by the City Government of Vienna, Austria in accordance with the Austrian Law and Guide for the Care and Use of Laboratory Animals as defined by the National Institute of Health, in accordance with the EU directive 2010/63/EU. Sciatic nerves of adult male Sprague Dawley rats were dissected and kept in prechilled phosphate buffered saline (PBS; PAA, Austria) on ice until further use, but not longer than 1 h. Schwann cells were isolated from sciatic nerves as previously described.^20^ Until further use cells were cultured in Dulbecco's modified Eagle medium (DMEM)-D-valine (PAA), supplemented with 10% FCS, 2 mM L-glutamine (PAA), 1% antibiotics (PAA), N_2_ supplement (Invitrogen, Germany), 10 μg/mL bovine pituitary extract, 5 μM forskolin, and on poly-L-lysine coated tissue culture polystyrene (TCPS).

### Preparation of fibrin and collagen substrates

For fibrin substrates prewarmed fibrinogen (TISSEEL; Baxter) was reconstituted in aprotinin, and thrombin 4 IU (Artiss; Baxter) in calcium chloride, according to the manufacturer's instructions. Fibrinogen and thrombin (both diluted 1:4 in DMEM high glucose) were mixed to equal parts, transferred into well plates (40 μL in 96-well plate), and left to form a gel at 37°C for 15 min.

For collagen substrates eight parts of type I rat tail collagen (2 mg/mL in 0.6% acetic acid; First Link, UK) were mixed on ice with one part of 10× minimal essential medium and one part DMEM high glucose. Mixture was neutralized using sodium hydroxide, transferred into well plates (40 μL per well in 96-well plates), and left to form a gel at 37°C for 15 min.

### Proliferation assay

Proliferation of Schwann cells on fibrin and collagen substrates was assessed using a 5-bromo-2-deoxyuridine (BrdU) uptake assay (Cell Proliferation Enzyme-Linked Immunosorbent Assay [ELISA] Kit; Roche Diagnostics, Basel, Switzerland) according to manufacturer's instructions. Twenty four and 72 h after seeding Schwann cells onto the substrates (7.5 × 10^3^ cells per well of a 96-well plate) or poly-L-lysine coated TCPS, respectively, 100 μmol/L BrdU was added, and cells were incubated for 24 h at standard cell culture conditions (37°C and 5% CO_2_). Subsequently culture plates were fixed with FixDenat solution and incubated with anti-BrdU peroxidase (POD) antibody solution for 60 min at room temperature. After washing the plate with PBS twice, tetramethylbenzidine was added for 30 min as a substrate. The reaction was stopped with 1 mol/L H_2_SO_4_, and absorption was measured at 450 with 690 nm as reference wavelength on an automatic microplate reader (Tecan Sunrise).

### Fabrication of stabilized collagen-fibrin blend gels

Collagen-fibrin blend gels were prepared as follows: 10× MEM and Collagen I (rat tail; First Link) were mixed on ice in a ratio of 1:8. Fibrinogen reconstituted in aprotinin was added to the solution v/v ratios of 10%, 20%, 30%, and 40%. Subsequently mixture was neutralized with 7% v/v sodium hydroxide. One unit thrombin in CaCl_2_ was added to polymerize fibrinogen. Two hundred forty microliter of the resulting collagen-fibrin solution was pipetted into 96-well plates and placed at 37°C. Gels were stabilized using RAFT absorbers (Lonza, Germany) for 15 min. Original height before and after stabilization, as well as potential swelling of the gels in 300 μL medium, was evaluated with side elevation view imaging (KSV CAM 200) on day 0, 1, 3, and 5.

### Mechanical testing of stabilized collagen and collagen-fibrin blend gels

Mechanical testing was performed on a Bose ElectroForce (3200 Series III) using WinTest 7 Software. Collagen and collagen-fibrin blend (10%, 20%) test specimens were prepared to be 20 mm in length, 3 × 0.3 mm at the ends and 2 × 0.3 mm in the middle, and were placed between the instrument grips with a gauge length of 12 mm. Testing was performed at 23°C, applying a load of 0.2 g/s until specimen failure. Data were plotted as stress/strain curves, and yield stress, as well as Young's modulus (linear region), was calculated.

### Viability of Schwann cells in stabilized collagen-fibrin gels

Survival of SCL4.1/F7 cells (Health Protection Agency, UK) seeded in stabilized collagen-fibrin blend gels was evaluated using MTT assay on day 0, 1, and 3 after stabilization. F7 SCL4 cells on TCPS acted as viability control. Stabilized gels were incubated with culture medium containing 650 μg/mL MTT [3-(4,5- dimethylthiazol-2-yl)-2,5-diphenyltetrazolium] bromide for 1.5 h at standard cell culture conditions (37°C, 5% CO_2_, and 80% humidity) (30 h after seeding). Medium was discarded, and MTT formazan precipitate was dissolved in 100 μL dimethylsulfoxide (DMSO) by shaking in dark for 1 h. Light absorbance at 540 nm was measured immediately, and optical density (OD) values were corrected for an unspecific background on an automatic microplate reader (Tecan Infinite M200).

### Engineered neural tissue

Collagen and collagen-fibrin blend gels (10%, 20%) containing SCL4.1/F7 cells (4 × 10^6^/mL) were cast in moulds (400 μL) containing tethering points at each end. Cells were left to align for 24 h before collagen gels, and collagen-fibrin blend gels were stabilized.^14^ Alignment of SCL4.1/F7 cells was evaluated using immunocytochemistry as previously described.^14,21^

### Neurite outgrowth assay

To assess potential effects of fibrin on neurite outgrowth, NG108 cells (3.5 × 10^4^ cells/cm^2^) were seeded on stabilized collagen gels, as well as collagen-fibrin blend gels (10%, 20%). Cells were left to adhere for 2 h in culture medium (DMEM high glucose, 10% FCS, 1% Pen/Strep). To stimulate neurite outgrowth, constructs were incubated in serum-free culture medium for 3 days. Constructs were rinsed in PBS, fixed in 4% (w/v) paraformaldehyde at 4°C for 24 h, and subsequently stained for βIII-Tubulin and Hoechst as previously described.^14^ Four equivalent fields per construct were analyzed using a fluorescence microscope (Zeiss Axio Lab A1) and ImageJ software.

### *In vivo* experiments

All experimental procedures involving animal surgery were conducted in accordance with the UK Animals (Scientific Procedures) Act (1986) and EU Directive 2010/63/EU and approved by the UCL Animal Welfare and Ethics Review Board.

### Preparation of EngNT constructs for implantation

Stabilized collagen and collagen-fibrin (10%) EngNT-Schwann cell constructs were cast in moulds as described above and left to adhere for 24 h. Subsequently constructs were thoroughly washed in PBS, cut to 8 mm length, and rolled parallel to the axis of cellular alignment to form a tight rod. Each rod, consisting of a 8 × 3 × 0.3 mm collagen sheet contained 4 × 10^5^ SCL4.1/F7 cells, was placed inside a 10 mm silicone tube (Syndev; 1.57 mm inner diameter, 0.42 mm wall thickness) and held in place using fibrin gel (TISSEEL, Baxter; diluted in DMEM 1:10). Final constructs were kept in Cryo-SFM medium (PromoCell, UK) until implantation *in vivo*.

### Rat sciatic nerve repair model

Sprague Dawley rats (200–250 g) were deeply anesthetized by inhalation of isoflurane, and anesthesia was maintained throughout the surgery through a mask. Analgesia (25 mg/kg Carprofen; Rimadyl^®^, Pfizer, UK) was administered subcutaneously. The sciatic nerve was exposed at mid-thigh level and transected to create an 8 mm gap. In both EngNT groups, the conduit was implanted by insertion of the proximal and distal nerve stumps into the 10 mm tube and coaptated to the conduit by two epineurial sutures (Ethilon 10/0; Ethicon-Johnson & Johnson, Brussels, Belgium) at each stump. Subsequently the wound was closed in layers, and animals were maintained for 4 weeks. After this period, animals were culled using CO_2_ asphyxiation, and their nerves were harvested under the dissecting microscope and fixed using 4% paraformaldehyde.

### Evaluation of axonal regeneration

After fixation with paraformaldehyde, harvested nerves were washed in PBS and cryoprotected in 30% sucrose in PBS overnight. Subsequently nerves were placed in optimum cutting temperature medium (OCT) compound, and transverse semi-thin cryostat sections were cut from the mid part of the device, as well as from the proximal and distal nerve stumps. Sections were blocked with 5% goat serum (15 min) and incubated with mouse monoclonal anti-200 kDa neurofilament antibody (1:1000; Abcam, UK) overnight at 4°C, followed by incubation with DyLight 546 horse anti-mouse immunoglobulin secondary antibody for 45 min (Vector Laboratories, Burlingame, CA). Axons were counted in a blinded manner from at least two sections per specimen using a Zeiss Axio Lab A1 fluorescence microscope and 40× magnification.

### Statistical analysis

All data in this study are shown as mean ± standard deviation. Statistical analysis was performed using Student *t*-test or one-way ANOVA (analysis of variance) followed by Tukey range test for significant differences between the means. Significance was considered for *p* < 0.05. For statistical calculations GraphPad Prism 5 for Mac OS X, Version 5.0b (GraphPad Software, Inc.) was used.

## Results

### Increased Schwann cell proliferation on fibrin substrates

**FIG. 1.**
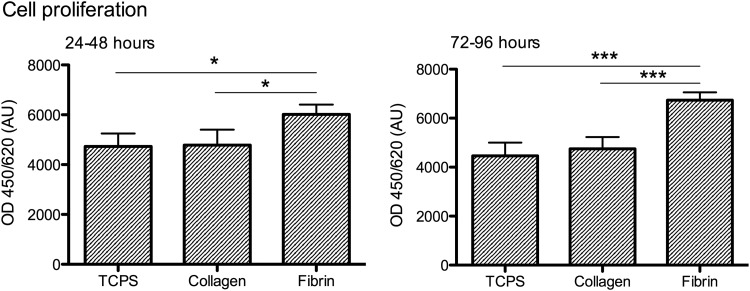
BrdU cell proliferation assay revealed a significantly higher proliferation rate of Schwann cells cultured on fibrin; BrdU assay performed 24 and 72 h after cell seeding; *n* = 4 different Schwann cell cultures, values were obtained in quadruplicates; statistical significance was tested with 1-way ANOVA and Tukey range test; data are presented as mean ± SD; *p < 0.05, ***p < 0.001. ANOVA, analysis of variance; BrdU, 5-bromo-2-deoxyuridine; SD, standard deviation.

Proliferation of primary rat Schwann cells cultured on fibrin and collagen substrates was evaluated compared to proliferation on poly-L-lysine coated TCPS using BrdU assay. As shown in Figure 1, an immediate effect of fibrin could be observed in the proliferation period between 24 and 48 h, showing a significant increase in proliferation of 25.7% compared with collagen and 26.2% compared with TCPS. Furthermore, prolonged culture (proliferation period between 72 and 96 h) revealed a sustained effect of fibrin on Schwann cell proliferation, resulting in an increase of 43.2% compared with collagen and 51.0% compared with TCPS.

### Fibrin concentration affects RAFT stabilization of collagen-fibrin blend gels

**FIG. 2.**
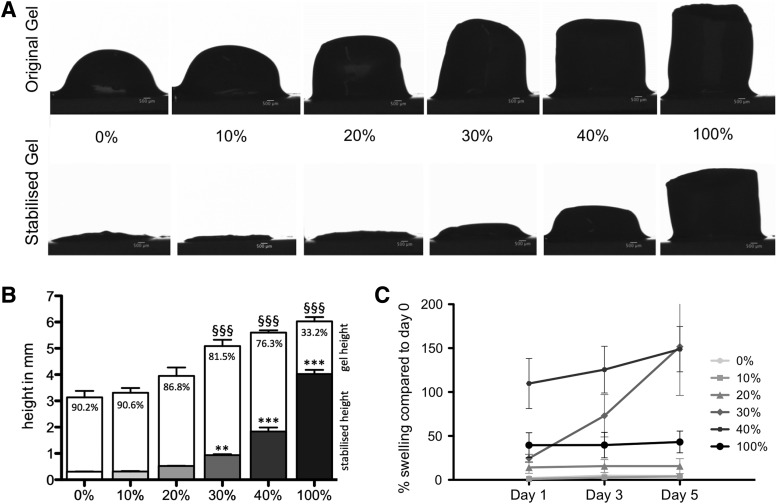
**(A)** Side view images before (original gel) and after (stabilized gel) removal of interstitial fluid of collagen gels containing 10%, 20%, 30%, and 40% fibrin, as well as a collagen control (0%) and a fibrin control (100%); micron bar = 500 μm; **(B)** height of the original gels (*white bars*) and stabilized gels (*gray bars*) in mm; percentage of height reduction after stabilization; data are presented as mean ± SD; significance tested with 1-way ANOVA and Tukey's posttest; ^§§§^*p* < 0.001 compared to 0% fibrin gel; **p < 0.01, ***p < 0.001 compared to stabilized 0% fibrin gel; *n* = 5; **(C)** swelling of the gels in percent compared to day 0 on day 1, 3, and 5; *n* = 5.

Figure 2A shows side elevation views of fully hydrated and stabilized collagen-fibrin gels compared to collagen gels and fibrin gels. A reduction in height of >85% was observed for collagen gels, as well as for the gels with lower fibrin concentrations (10%, 20%), resulting in a final gel height of <0.5 mm (Fig. 2B). Gels with higher concentrations of fibrin (30%, 40%, and 100%) could not be compressed to this extent, as seen in Figure 2B (reduction in mean height of 81.5–33.2%), where the final mean height of the compressed gels was 0.93–4.02 mm. Fibrin concentrations above 30% showed a significantly increased height of the original gel, as well as RAFT stabilized gels. Height fluctuation in culture medium after RAFT stabilization was assessed at several time points (Fig. 2C). Fibrin concentrations below 30% were not subject to extensive swelling, while 30% and 40% fibrin revealed continuous swelling over a time period of 5 days.

### Mechanical properties alter with increasing fibrin concentration

**FIG. 3.**
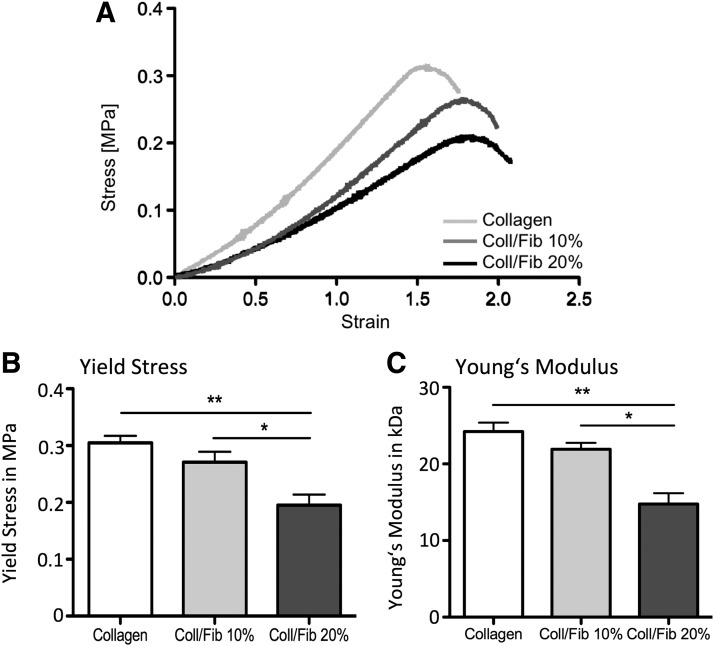
Mechanical testing of stabilized collagen gels, or 10% and 20% collagen-fibrin (Coll/Fib) blend gels; Representative stress–strain curves **(A)**, yield stress **(B)**, and Young's Modulus **(C)**; *n* = 4; data are presented as mean ± SD; significance tested with 1-way ANOVA and Tukey's posttest; *p < 0.05, **p < 0.01.

As the 30% and 40% collagen-fibrin blend gels could not be fully stabilized and furthermore showed extensive swelling, no further tests were conducted with these concentrations. Tensile testing of stabilized collagen gels, as well as collagen-fibrin blend gels, is shown in Figure 3. Stabilized collagen gels revealed a yield strength of 0.30 ± 0.02 MPa and a Young's Modulus of 24 ± 2.1 kPa. Addition of 10% fibrin revealed no significant changes in yield strength (0.27 ± 0.2 MPa) and Young's Modulus (22 ± 2.3 kPa), while addition of 20% fibrin led to significant decrease of both, yield strength (0.19 ± 0.018 MPa), as well as Young's Modulus (15 ± 1.2 kPa).

### Increased viability, alignment, and neurite outgrowth on collagen-fibrin gels

**FIG. 4.**
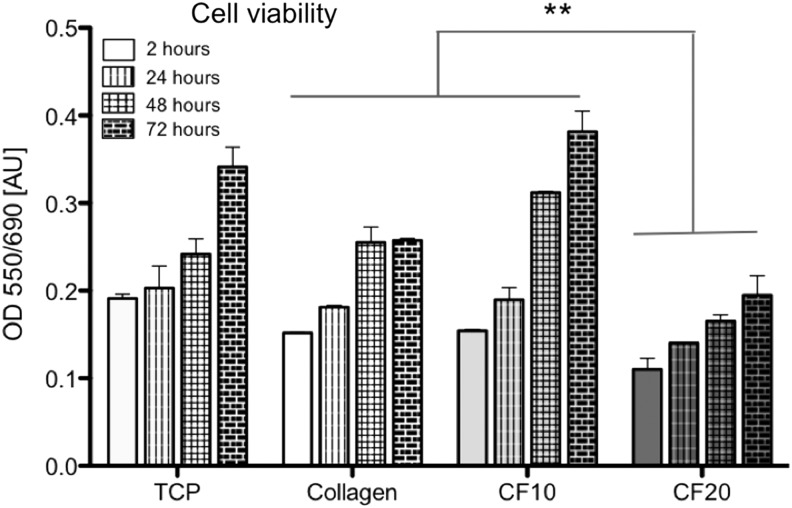
Cell viability of SCL4.1/F7 cells in stabilized collagen gels, or 10% and 20% collagen-fibrin blend gels, assessed with MTT assay on day 0, 1, 2, and 3 after stabilization compared to SCL4.1/F7 cells seeded on TCPS; data are presented as mean ± SD; **p < 0.01; significance tested with 1-way ANOVA and Tukey's posttest; *n* = 4. TCPS, tissue culture polystyrene.

Viability of SCL4.1/F7 cells in stabilized collagen-fibrin gels was assessed with MTT assay over 72 h (Fig. 4). At every time point (2, 24, 48, and 72 h after stabilization), cells in 20% fibrin blend gels showed significantly decreased viability compared to 10% fibrin blend gels, collagen gels, and TCPS. Over time viability in 20% fibrin blend gels gradually increased, indicating that the proliferative capacity of the cells remains unaffected. Interestingly, on TCPS, as well as in 10% fibrin blend gels, cell viability strongly increased between 48 and 72 h (39.7% and 29.3%, respectively), while viability in the collagen gel remained at the same level.

**FIG. 5.**
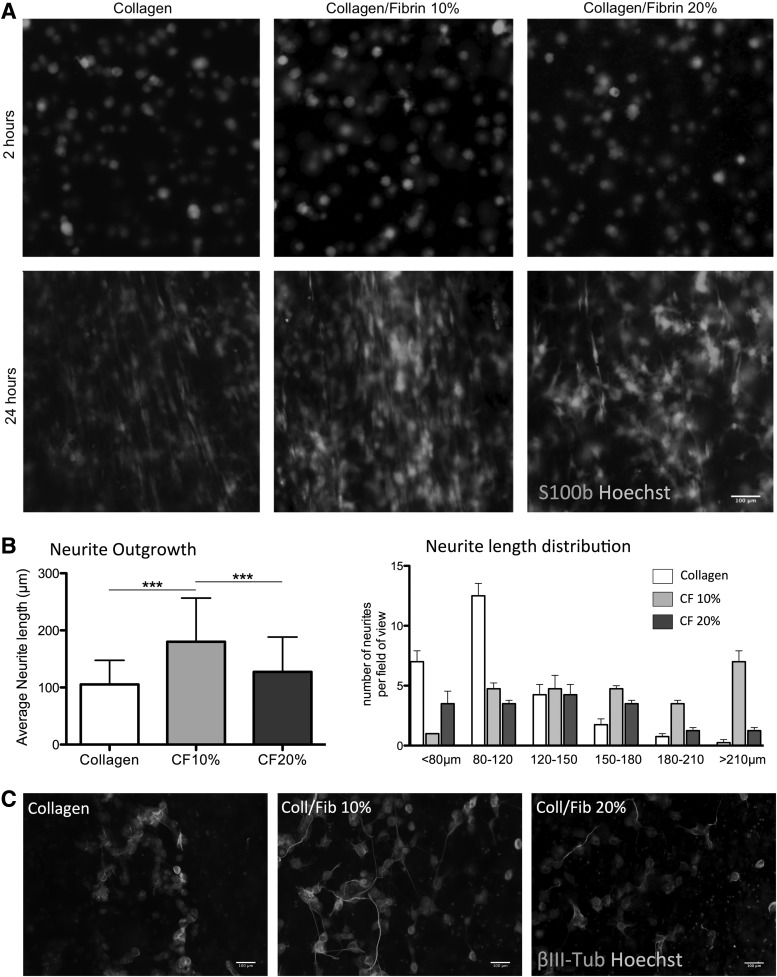
**(A)** Fluorescence micrographs depicting SCL4.1/F7 cells stained with S100b and Hoechst in a stabilized collagen EngNT and collagen-fibrin EngNt (10%, 20% fibrin), respectively; *upper panel* shows cells 2 h after cell seeding, *lower panel* 24 h after cell seeding; **(B)** Average neurite length of NG108 cells after 72 h on collagen EngNT and collagen-fibrin EngNT (10%, 20%), and size distribution of neurites per field of view; four different areas per EngNT were analyzed, *n* = 5; data are presented as mean ± SD; ***p < 0.001; significance tested with 1-way ANOVA and Tukey's posttest; **(C)** Fluorescence micrographs depicting NG108 cells stained with beta-3-tubulin and Hoechst after 72 h on collagen EngNT and collagen-fibrin EngNT (10%, 20%). EngNT, engineered neural tissue.

Figure 5A shows SCL4.1/F7 cells in a tethered stabilized EngNT 2 and 24 h after cell seeding. Two hours after cell seeding cells exhibited a round shape in all groups (collagen, collagen-fibrin blend gel 10% and 20%). After 24 h cells in collagen gels and 10% collagen-fibrin blend gel adopted a bipolar elongated shape predominantly in parallel to the long axis of the gel. In contrast SCL4.1/F7 cells in the 20% collagen-fibrin blend gel were predominantly randomly organized and did not show alignment.

To assess potential effects of fibrin on neurite outgrowth, NG108 cells were cocultured on EngNT made from collagen and collagen-fibrin blends (10%, 20%), respectively. After 72 h the mean neurite length assessed for neurites grown on collagen constructs was 105.3 ± 42.0 μm. As shown in Figure 5B, the inclusion of 10% fibrin significantly increased mean neurite length by 68.2% (180.6 ± 76.5 μm) compared to neurites grown on collagen EngNT. This enhancing effect was not observed for 20% fibrin EngNT (127.4 ± 61.0 μm). Average length distribution analysis revealed that neurites grown on collagen EngNT showed neurite length peaks around 80 μm and between 80 and 120 μm, while 10% fibrin shifts this peak toward longer neurites (>150 μm).

### Fibrin increases axonal regeneration *in vivo*

**FIG. 6.**
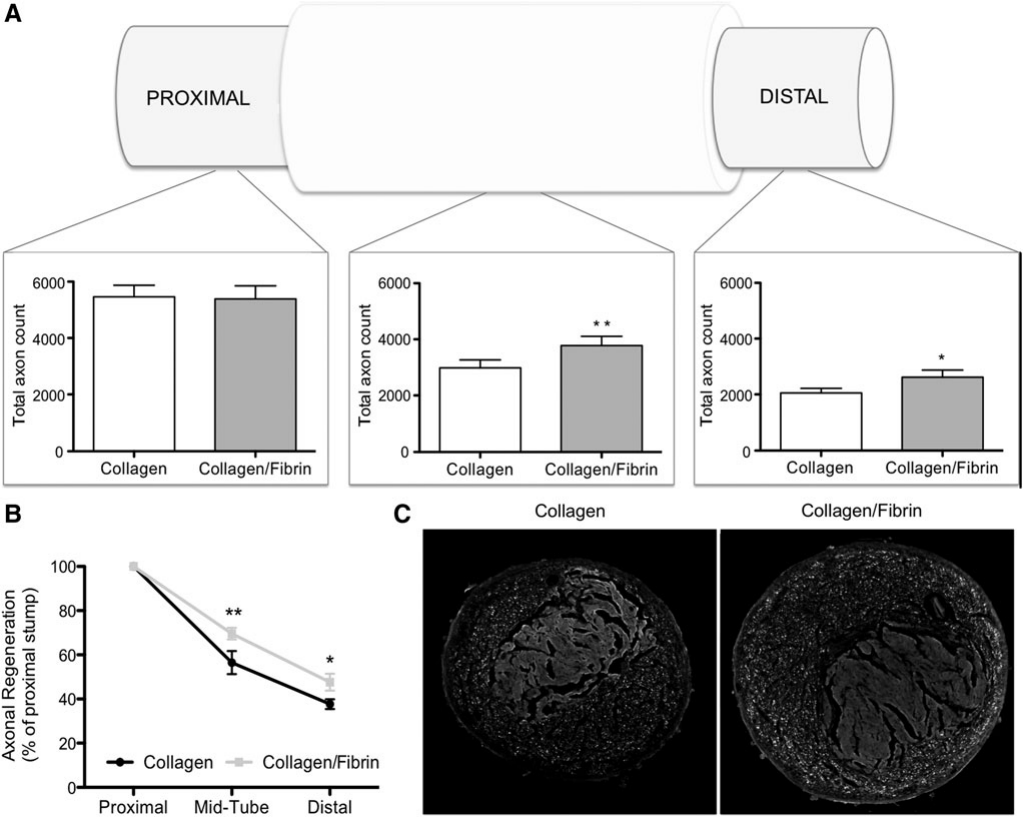
Evaluation of axonal regeneration through an 8 mm silicone tube containing collagen or collagen-fibrin EngNT. **(A)** Total axon count proximal to the device, mid-tube, and distal to the device; **(B)** Regeneration relative to proximal stump; **(C)** representative images of transversal sections (*n* = 5; data are presented as Mean ± SD; *p < 0.05, **p < 0.01; significance tested with Student *t*-test.

Subsequent to *in vitro* testing of several collagen-fibrin blends, the most promising candidate was tested in a rat sciatic nerve repair model. Four weeks after bridging an 8 mm sciatic nerve defect in the rat, nerves were explanted and axons were counted after immunohistochemical staining of transverse sections for 200 kDa neurofilament, proximal and distal to the tube, as well as in the middle section of the device. As seen in Figure 6, proximal to the injury no significant differences between the two groups could be detected (collagen: 5464.3 ± 408.5 axons; collagen-fibrin: 5386.2 ± 464.6 axons). Analyses of the device midsection revealed a significantly higher axon count in the collagen-fibrin blend group (3738 ± 370 axons, 68.73% of the proximal stump) compared to the collagen group (2985 ± 281 axons, 56.03% of the proximal stump). This significant difference continues also distal to the device with an axon count of 2558 ± 361 axons in the collagen-fibrin blend group (47.59% of the proximal stump) compared to 2053 ± 162 axons in the collagen group (37.38% of the proximal stump).

## Discussion

The study presented focuses on improving EngNT technology, an approach developed for the construction of living artificial nerve tissue that mimics key features of the nerve graft and has previously been made using type I collagen. Collagen has been proven to be a suitable and versatile endogenous biomaterial for peripheral nerve regeneration, being used as a tube material,^22^ as well as luminal filler in various forms, and has been shown to be compatible with delivery of Schwann cells.^23,24^ While collagen-based EngNT has been used to deliver a range of potential cell therapies,^25,26^ for long-distance defects Schwann cells (or Schwann cell-like therapeutic cells) have to remain in a pro-regenerative proliferative state over a prolonged period of time. Several studies have shown that fibrin increases the proliferative activity of Schwann cells, pushing them into the pro-regenerative phenotype by binding with integrin αVβ8.^27^ This interaction is activating the MAP-Kinase pathway, resulting in decreased myelination and increased expression of p75NGFR, which are associated with Schwann cell proliferation, as well as c-Jun, a marker for regenerative Schwann cells.^28^ In this study, we were able to confirm that cultivation of primary rat Schwann cells on fibrin led to significantly increased proliferative activity *in vitro*, compared to cultivation on collagen type I and tissue culture plastic coated with poly-L-lysine on an early time point (48 h), as well as on a later time point (96 h).

To explore whether these positive characteristics of fibrin could be used to improve collagen-based tissue engineered constructs, it was incorporated into collagen gels at a broad range of different concentrations. Increasing the amount of fibrin resulted in overall bulkier gels, reflected in initial height, which was significantly greater in the 30%, 40%, and 100% fibrin group, compared to collagen. This effect may be explained by altered polymerization dynamics with increasing fibrin content, shifting from a collagen-co-gel, where polymerization is predominantly driven by the collagen gel formation, to a fibrin-co-gel with the vice versa effect, as previously described by Lai *et al.*^29^

In addition to altered characteristics of the starting gels, in blend gels with higher fibrin content (30%, 40%, and 100%) interstitial fluid was not removed to the same extent as in collagen-dominated gels during the stabilization step, resulting in gels with an overall increased height. Behavior in culture medium was observed over several days: stabilized collagen constructs, as well as 10% and 20% fibrin blend gels, did not exhibit significant signs of swelling. However, blend gels with higher fibrin contents (30%, 40%, and 100%) were subject to extensive swelling by reuptake of medium. This effect is likely to be the result of a reduced number of collagen fibrils and the resulting reduction in interactions between them, which could reduce stability. As shown in Figure 3, yield stress, as well as Young's modulus, was significantly decreased in the 20% fibrin group, while 10% fibrin blend gels showed no significant difference compared to collagen gels tested.

Interestingly, increasing the proportion of fibrin from 10% to 20% in gels containing SCL4.1/F7 Schwann cells altered their characteristics in such a way that alignment of the cells and subsequent support of neurite growth *in vitro* were reduced. In addition, cells appear to be less viable in 20% fibrin gels compared to 10% fibrin or collagen gels. Concerning alignment it is likely that the addition of 20% fibrin (or 18 mg/mL) to collagen gels inhibited or delayed the modulation not only due to increased stiffness but also overall denser structure of fibrin. Ho *et al.*^30^ showed in their 2006 study that fibrin clot structure changes significantly with increasing fibrinogen content, leading to a more dense and less regular structure of fibrin (5 mg/mL compared to 17 mg/mL fibrinogen).^31^ In addition to reduced Schwann cell alignment, the presence of increased fibrin concentrations may have reduced neurite extension directly, as indicated by previous studies.^32^

Having identified 10% fibrin 90% collagen as an optimal EngNT material formulation that promoted neuronal regeneration *in vitro* to a greater extent than collagen-only EngNT, an *in vivo* comparison was conducted. While this was a limited study with short time duration and 8 mm gap it was clear that incorporating 10% fibrin in the EngNT increased the number of neurites present both within the repair and in the distal stump. This confirms the *in vitro* findings indicating that incorporation of 10% fibrin has beneficial effects and provides a promising first indication that modification of the material component of EngNT can be conducted in this way. An increase in the number of regenerating neurites crossing into the distal stump at 4 weeks has the potential to result in more robust functional recovery, and therefore, this approach should be investigated further. In particular, the implication that the increased regeneration support shown in this study is due to the effects of fibrin on Schwann cell behavior means it will be important to test this approach in a longer “critical sized” gap model where the presence of aligned Schwann cells is of greater significance.^33^ Furthermore, it will be interesting to investigate whether the addition of 10% fibrin to EngNT is beneficial in promoting regeneration when other types of cells are used rather than Schwann cells. In particular, therapeutic cells such as those derived from stem cells provide opportunities for clinical translation of tissue-engineered nerve constructs as alternatives to the autograft,^34^ and the methods established in this study provide a suitable basis for investigating this further. The widespread availability and use of clinical-grade fibrin products such as the material used in this study also support the feasibility of incorporating it into EngNT without complicating subsequent manufacturing and regulatory requirements.

In conclusion this study has demonstrated that fibrin can be blended with collagen to generate stable aligned EngNT constructs containing Schwann cells. These blended engineered tissues were able to improve Schwann cell viability and promoted neuronal growth both *in vitro* and *in vivo* to a greater extent than equivalent constructs made using collagen without fibrin. The mechanical properties and stability of a range of novel collagen-fibrin blend formulations were characterized in detail along with their effect on cell viability, providing new insights into how these natural biomaterials can be combined for future tissue engineering applications.
